# Modelling why 70 per cent of the world’s population lack access to surgery

**DOI:** 10.1093/bjs/znad005

**Published:** 2023-02-08

**Authors:** Niclas Rudolfson, Mark G Shrime, Blake C Alkire

**Affiliations:** Surgery and Public Health, Department of Clinical Sciences Lund, Lund University, Lund, Sweden; Mercy Ships, Garden Valley, Texas, USA; Department of Global Health and Social Medicine, Harvard Medical School, Boston, Massachusetts, USA; Department of Global Health and Social Medicine, Harvard Medical School, Boston, Massachusetts, USA; Center for Global Surgery Evaluation, Massachusetts Eye and Ear Infirmary, Boston, Massachusetts, USA

*Dear Editor*

The Lancet Commission on Global Surgery^1^ showed that at least 140 million additional surgical procedures are needed to prevent loss of life or significant disability worldwide, and that implementing surgical, obstetric and anaesthesia care is a cost-effective solution for global public health. The commission set out to estimate the proportion of the global population that lack access to such care and, as a first step, the meaning of the word access needed to be clearly defined.

Previous research^2^ had used operating theatre density as a proxy for surgical capacity and found that 2 billion people lacked access to surgery. It is however well known in the sustainable development goals era, infrastructure to deliver healthcare is not sufficient to achieve universal healthcare coverage. As research in high-income settings has consistently shown, there is a critical window of time between onset of symptoms and receiving treatment for many surgical conditions. If patients are unable to access surgical care during this time frame, they are much more likely to suffer from a poor outcome (or even death). Globally, more people are estimated to die as a result of inadequate quality of care rather than availability^3^, i.e. care must be safe. Finally, as healthcare expenses are one of the leading causes of impoverishment^4^, wreaking havoc upon entire households, surgical care must also be affordable. Any definition of meaningful access consequently needs to address not only capacity but also the timeliness, safety, and affordability of the care provided.

Using the above four axes of access, Alkire *et al*.^5^ estimated the proportion of each country’s population that did have access to surgical care (a function of the joint probability of having access to each of the axes of access), ultimately determining that up to 70 per cent of the world’s population lacks full access to surgical care.

For this letter, the methodology is revisited but applied to every possible combination of joint probabilities, and by doing so the relative scale of the issues that need to be solved in order to achieve universal healthcare in surgery is determined (*Fig. 1* and *Table S1*). The global population that lacks access to surgery owing to a combination of one or more factors is presented as a set of area-proportional ellipses in an Euler diagram.

**Fig. 1:**
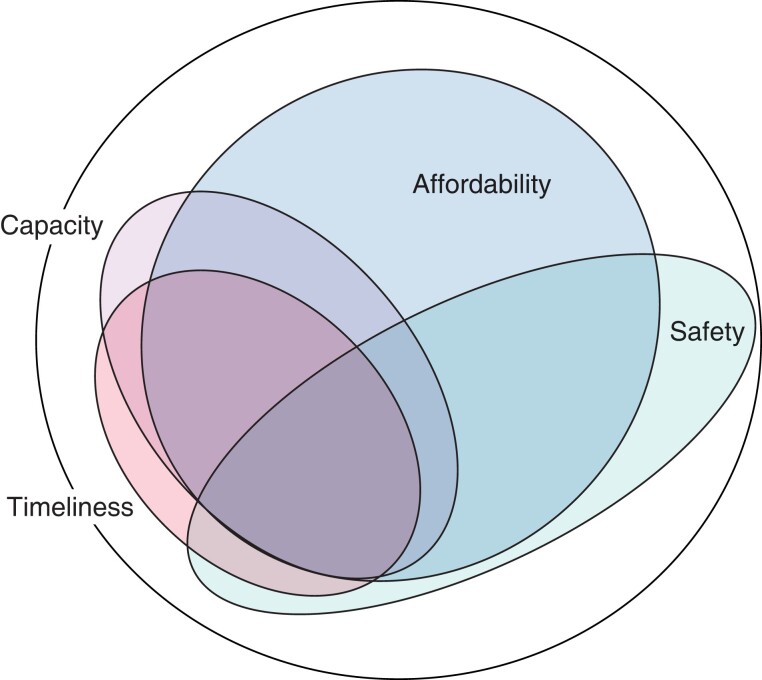
Area-proportional Euler diagram of the lack of access to surgery The large circle represents the total global population, and the smaller ellipses represent those who do not have access to surgical care owing to each factor.

What are the lessons to be learnt from such a mathematical exercise? By visualizing data in this way, the multidimensional nature of the problem immediately becomes obvious. Of the billions of people estimated to lack access to surgery, 60 per cent do so because a lack of two or more factors, 27 per cent due to a lack of three or more factors, and 13 per cent owing to all four.

The relative importance of the different factors can thus be understood, in particular the outsized influence of affordability on the number of people who lack full access to surgery. In the authors’ experience, much of the foreign aid for surgery is aimed primarily at capacity building, whereas comparatively less attention is given to affordability. Conceivably, when global health programmes are run by teams comprising mainly physicians and other health workers, issues requiring entirely different skill sets, such as insurance schemes or road networks, are not prioritized. Understanding this can aid in moving towards universal health coverage as a continuum rather than a binary headcount, where solutions will have to be tailored to the specific hindrance rather than one size fits all.

In light of these lessons, any attempt to mitigate this ongoing public health crisis will need to be focused not solely on operating capacity, but will need to be as multifactorial as the problem it seeks to solve.

## Supplementary Material

znad005_Supplementary_Data

## Data Availability

Data utilized in the study are available from the corresponding author upon reasonable request.
