# Supplementary material for: Modelling why 70 per cent of the world’s population lack access to surgery
Source: Br J Surg. 2023 Feb 8;110(4):500–1. doi: 10.1093/bjs/znad005 (PMC10364489; doi:10.1093/bjs/znad005)
Supplement: znad005_Supplementary_Data [file znad005_supplementary_data.docx]

**Title*: Why does 70% of the world’s population lack access to surgery?***

**Authors and affiliations**

*Niclas Rudolfson*

Centre for Surgery and Public Health, Department of Clinical Sciences Lund, Lund University, Sweden

*Mark G. Shrime*

Mercy Ships, Garden Valley, Texas, USA

Department of Global Health and Social Medicine, Harvard Medical School, Boston, MA, USA

*Blake C. Alkire*

Center for Global Surgery Evaluation, Massachusetts Eye and Ear Infirmary, Boston, MA, USA

Department of Global Health and Social Medicine, Harvard Medical School, Boston, MA, USA

**Corresponding author**

Niclas Rudolfson, Centre for Surgery and Public Health, Pediatrics, Department of Clinical Sciences Lund, Lund University, Lund, Sweden

Postal Adress: Sölvegatan 19, 221 84 Lund, Sweden

Email: [Niclas.rudolfson@med.lu.se](mailto:Niclas.rudolfson@med.lu.se), Phone number: +46 735 034 538

**Supplementary Materials - Index**

| **Supplementary Figures and Tables** |  |
| --- | --- |
| Table S1 | *pag. 2* |
|  |  |

**Supplementary Figures and Tables**

**Table S1**. The estimated number of people globally that lack access to surgery due to issues with timeliness (T), capacity (C), affordability (A), safety (S), and the combinations thereof. *

| Factor | Million people |
| --- | --- |
| T | 1,500 |
| C | 1,900 |
| S | 2,400 |
| A | 3,800 |
| T ∪ C | 2,200 |
| T ∪ S | 3,200 |
| T ∪ A | 4,100 |
| C ∪ S | 3,400 |
| C ∪ A | 4,100 |
| S ∪ A | 4,500 |
| T ∪ C ∪ S | 3,600 |
| T ∪ C ∪ A | 4,300 |
| T ∪ S ∪ A | 4,700 |
| C ∪ S ∪ A | 4,700 |
| T ∪ C ∪ S ∪ A | 4,800 |

*Global population used in these calculations: 7 billion.
